# Monocyte differentiation and macrophage priming are regulated differentially by pentraxins and their ligands

**DOI:** 10.1186/s12865-017-0214-z

**Published:** 2017-06-15

**Authors:** Darrell Pilling, Elkin Galvis-Carvajal, Tejas R. Karhadkar, Nehemiah Cox, Richard H. Gomer

**Affiliations:** Department of Biology, Texas A&M University, 3474 TAMU, College Station, TX 77843-3474 USA

**Keywords:** Pentraxins, Inflammation, Monocyte, Macrophage, C1q, SAP, CRP, PTX3, Factor H, Mannose binding lectin, MBL

## Abstract

**Background:**

Circulating bone marrow-derived monocytes can leave the blood, enter a tissue, and differentiate into M1 inflammatory, M2a remodeling/fibrotic, or M2c/Mreg resolving/immune-regulatory macrophages. Macrophages can also convert from one of the above types to another. Pentraxins are secreted proteins that bind to, and promote efficient clearance of, microbial pathogens and cellular debris during infection, inflammation, and tissue damage. The pentraxins C-reactive protein (CRP), serum amyloid P (SAP), and pentraxin-3 (PTX3) can also bind a variety of endogenous ligands. As monocytes and macrophages are exposed to differing concentrations of pentraxins and their ligands during infection, inflammation, and tissue damage, we assessed what effect pentraxins and their ligands have on these cells.

**Results:**

We found that many polarization markers do not discriminate between the effects of pentraxins and their ligands on macrophages. However, pentraxins, their ligands, and cytokines differentially regulate the expression of the hemoglobin-haptoglobin complex receptor CD163, the sialic acid-binding lectin CD169, and the macrophage mannose receptor CD206. CRP, a pentraxin generally thought of as being pro-inflammatory, increases the extracellular accumulation of the anti-inflammatory cytokine IL-10, and this effect is attenuated by GM-CSF, mannose-binding lectin, and factor H.

**Conclusions:**

These results suggest that the presence of pentraxins and their ligands regulate macrophage differentiation in the blood and tissues, and that CRP may be a potent inducer of the anti-inflammatory cytokine IL-10.

**Electronic supplementary material:**

The online version of this article (doi:10.1186/s12865-017-0214-z) contains supplementary material, which is available to authorized users.

## Background

Cells of the mononuclear phagocytic system, monocytes and macrophages, are found in every tissue of the body and regulate infections, inflammation, and tissue repair, and are critical in the protection from, or development of, autoimmune diseases, asthma, fibrosis, and cancer [1]. Tissue resident macrophages derive from progenitor cells that develop in the fetal yolk sac and fetal liver, whereas circulating monocytes are bone marrow-derived cells that leave the blood, enter tissues, and then differentiate into macrophages during inflammation, infection, or tissue damage [2]. There are different types of macrophages such as M1 inflammatory macrophages, and M2 remodeling/fibrotic (M2a) or resolving/immune-regulatory (M2c; sometimes called Mreg) macrophages [3]. Although many markers have been proposed that discriminate these subsets, there are no definitive markers to identify macrophage subtypes [4]. In addition, as macrophages change their phenotypic markers and physiology when exposed to different environmental signals, macrophage phenotypes may be more of a series of overlapping subsets or a continuum, rather than defined and permanent subsets [5].

In persistent diseases, macrophages can be either activated to drive a disease process, or either absent or suppressed and therefore unable to aid in the resolution of a condition [1]. In tuberculosis, Leishmaniasis, trypanosome infections, and some tumors, the macrophages have an M2a or M2c phenotype, and it has been hypothesized that shifting these to an M1 phenotype could be therapeutic [1]. Conversely, in fibrosis, the macrophages have an M2a pro-fibrotic phenotype, and shifting these to an M2c phenotype could be therapeutic [1, 6]. Understanding, and being able to manipulate, macrophage differentiation could have a significant impact on a wide variety of diseases.

Pentraxins are secreted proteins that bind to, and promote efficient clearance of, microbial pathogens and cellular debris during infection, inflammation, and tissue damage [7]. Pentraxins also regulate macrophage responses. The pentraxin serum amyloid P (SAP) is a constitutive component of plasma and drives monocytes and macrophages to a M2c phenotype, as defined by upregulation of the potent anti-inflammatory and anti-fibrotic cytokine IL-10 [8–10]. In animal models, SAP inhibits fibrosis and promotes disease resolution by activating the CD209 receptor [11], activating Fcγ receptors (FcγR) [8, 11, 12] and by potentiating extracellular accumulation of IL-10 [6, 8, 11]. In contrast to SAP, the pentraxin CRP has been thought to induce a M1 phenotype [13]. Serum levels of human CRP increase up to a thousand fold during infection and inflammation [14], and elevated serum CRP levels are a biomarker for predicting inflammatory diseases [15]. In animals, overexpression of CRP strongly potentiates inflammation and fibrosis [16]. However, CRP can inhibit experimental allergic encephalomyelitis (EAE) and kidney inflammation by macrophage- and IL-10-dependent mechanisms [17, 18]. A third pentraxin, PTX3, is upregulated during inflammation in humans, but in mice appears to be pro-inflammatory in some models and limits inflammation in other models, and its effects on human or mouse macrophages is unclear [7, 19]. These data indicate that pentraxins have complex and important roles in inflammation and tissue damage. Pentraxins not only act on cells as independent molecules but also in association with a variety of ligands [7]. SAP, CRP, and PTX3 all bind the complement component C1q, and promote phagocytosis of complement-bound bacteria [20–22]. Additional pentraxin ligands include complement component Factor H, which binds CRP and PTX3, and mannose-binding lectin (MBL), which binds SAP and PTX3 [7, 23, 24].

As circulating monocytes, differentiating macrophages, and tissue resident macrophages are exposed to the three pentraxins and their ligands during infection, inflammation, and tissue damage, we assessed what effect pentraxins and their ligands have on macrophages. In this report, we show that pentraxins and their ligands have distinct effects on monocyte differentiation into macrophages and macrophage priming from one subtype to another subtype. In addition, we show that CRP can induce production of the anti-inflammatory cytokine IL-10.

## Methods

### Cell isolation and cell culture conditions

All protocols were approved by the local ethical committees and performed in accordance with national guidelines and regulations. Human peripheral blood was collected from healthy adult volunteers who gave written consent and with specific approval from the Texas A&M University human subjects Institutional Review Board. Peripheral blood mononuclear cells (PBMC) were isolated from heparinized blood using Ficoll-Paque Plus (GE Healthcare Biosciences, Piscataway, NJ), as described previously [9]. PBMC were cultured at 37 °C in a humidified incubator with 5% (vol/vol) CO_2_ in either 8-well glass slides (Falcon-Corning, Tewksbury, MA or EMD-Millipore, Billerica, MA) or 96 well μ-plates (ibidi, Madison, WI) with 200 μl/well at 5 x 10^5^ cells per ml in RPMI-1640 (Lonza, Walkersville, MD) containing 100 U/ml penicillin, 100 μg/ml streptomycin (Lonza), and 10% fetal calf serum (FCS; Seradigm, Radnor, PA) [9, 11, 25]. Although both human AB serum and FCS can be used for human monocyte/macrophage cultures, we used FCS as it contains low levels of pentraxins and their ligands [26–31].

### Monocyte differentiation, macrophage priming, and macrophage polarization

For monocyte differentiation, PBMC were incubated for 6 days in the presence or absence of the indicated concentrations of SAP (EMD Millipore, Billerica, MA), CRP (#30-ac05AF, Fitzgerald Industries, Acton, MA), or PTX3 (R&D Systems, Minneapolis, MN) [9, 25]. As commercial SAP preparations contain 0.1% azide, we buffer-exchange the SAP into 20 mM sodium phosphate, pH 7.4, as described previously [9, 32]. CRP and PTX3 preparations were purchased free of azide. For macrophage priming, PBMC were incubated in the presence or absence of 25 ng/ml M-CSF or GM-CSF (BioLegend, San Diego, CA) for 6 days [4, 33, 34]. The medium was then removed and fresh medium containing M-CSF or GM-CSF was then added, containing the indicated concentrations of pentraxins in the presence or absence of 30 μg/ml C1q (Fitzgerald Industries), 100 μg/ml factor H (Fitzgerald), or 2 μg/ml MBL (NovoProtein, Summit, NJ) for an additional 2 days. To polarize macrophages, PBMC were incubated for 6 days in the presence of 25 ng/ml M-CSF or GM-CSF and then polarized into M1 macrophages with 10 ng/ml IFN-γ (BioLegend) and LPS (Sigma, St. Louis, MO) or M2 macrophages with 10 ng/ml IL-4 (BioLegend) [33, 35, 36]. All reagents were isolated from human material and/or tested for LPS/endotoxin and found to be <0.01 EU/ml or <1.0 EU per μg of protein (apart from the LPS), as determined by the manufacturer. After 6-day monocyte differentiation experiments or 8-day macrophage priming experiments, supernatants were collected and stored at either 4 °C (for less than 2 days) or at −20 °C, and the plates were air-dried as described previously [9]. Supernatants were analyzed by ELISA using kits for IL-4 (Peprotech, Rocky Hill, NJ), IL-10 (BioLegend), IL-12 (BioLegend), and IFN-γ (Peprotech) following the vendor’s protocol.

### Immunocytochemistry (ICC)

For 8-well slides and 96 well plates, cells were fixed with acetone for 15 min, air dried for 15 min and then non-specific binding was blocked by incubation in PBS containing 4% BSA (PBS-BSA) for 60 min. Slides were then incubated with 5 μg/ml primary antibodies (Table 1) in PBS-BSA for 60 min as described previously [9]. Isotype-matched irrelevant mouse and rat monoclonal antibodies (BioLegend), or irrelevant rabbit or goat polyclonal antibodies (R&D Systems, Minneapolis, MN), at 5 μg/ml in PBS-BSA were used as controls. Primary antibodies were detected with either biotinylated donkey F(ab’)_2_ anti-mouse IgG, biotinylated mouse F(ab’)_2_ anti-rat IgG, or biotinylated donkey F(ab’)_2_ anti-rabbit IgG (all cross-adsorbed against human Ig; Jackson ImmunoResearch, West Grove, PA). All secondary antibodies were used at 1 μg/ml in PBS-BSA for 30 min. Biotinylated antibodies were detected by a 1/500 dilution of ExtrAvidin alkaline phosphatase (Vector Laboratories, Burlingame, CA) in PBS-BSA. Staining was developed with the Vector Red Alkaline Phosphatase Kit (Vector Laboratories) for 5–7 min, and then counterstained with Gill’s hematoxylin #3 (Sigma-Aldrich, St. Louis, MO) following the manufacturer’s directions. Macrophages were identified as 15–40 μm diameter cells with a large nucleus and pronounced cytoplasm.

**Table 1:** Antibody list

Marker	Description of marker	Clone or Catalog number	Isotype	Source
CD163	hemoglobin-haptoglobin complex receptor	RM3/1	Mouse IgG1	BioLegend
CD169	Siglec-1 Sialoadhesin (Sn)	7-239	Mouse IgG1	BioLegend
CD172a/b	Signal regulatory protein α/β (SIRPα/β)	SE5A5	Mouse IgG1	BioLegend
CD200R	OX-2 receptor-cell surface receptor	OX-108	Mouse IgG1	BioLegend
CD206	Macrophage mannose receptor (MMR)	15-2	Mouse IgG1	BioLegend
CCR7 (CD197)	Receptor for chemokines CCL19/ELC and CCL21	3D12	Rat IgG2a	eBioscience
Dectin-1 (CLEC7A)	C-type lectin family	Clone 259931 MAB 1859	Mouse IgG2b	R&D Systems
Fibronectin	Matrix protein	EP5	Mouse IgG1	GeneTex
IFN-γ receptor (CD119)	Cytokine receptor	GIR-94	Mouse IgG2b	BD Bioscience
Interferon regulatory factor 4 (IRF4)	transcription factor	EP5699 (ab133590)	Rabbit monoclonal	Abcam
Interferon regulatory factor 5 (IRF5)	transcription factor	EPR6094 (ab124792)	Rabbit monoclonal	Abcam
MHC class II (DR)	major histocompatibility complex	L243	Mouse IgG2a	BioLegend
Raf1	serine/threonine-protein kinase	Y198 (ab32025)	Rabbit monoclonal	Abcam
RELM Beta (FIZZ 2)	resistin-like molecule beta Found in inflammatory zone 2	GTX88677	Goat polyclonal	GeneTex
TNFRSF14 (CD270)	cell surface receptor of the TNF-receptor superfamily	ab47677	Rabbit polyclonal	Abcam
sphingosine kinase 1 (SPHK1)	phosphorylates sphingosine to sphingosine-1-phosphate	GTX107509	Rabbit polyclonal	GeneTex

### Statistics

Statistical analysis was performed using GraphPad Prism 4 software (GraphPad, San Diego, CA). Statistical significance between two groups was determined by t tests or Mann–Whitney tests, or between multiple groups using 1-way ANOVA with Dunn’s test. Significance was defined as *p* < 0.05.

## Results

### Pentraxins affect the expression of CD163, CD169, and CD206 in macrophages

**Fig. 1:**
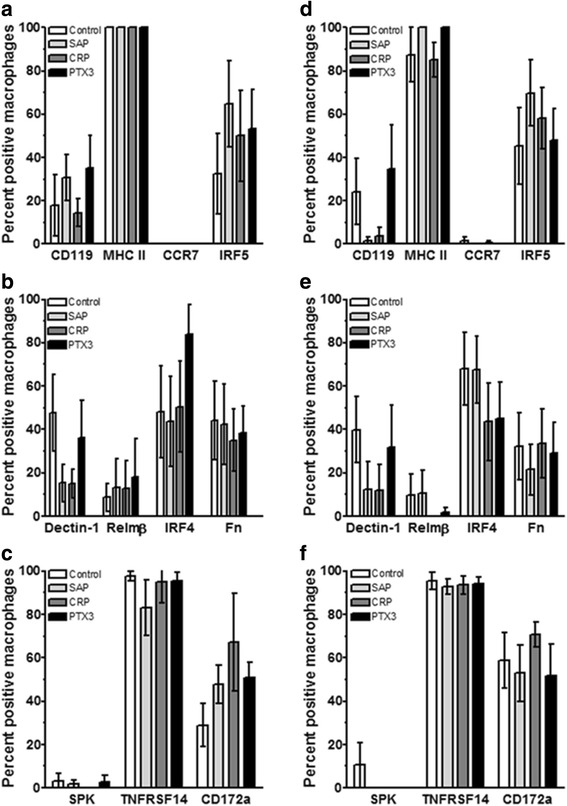
Expression of M1/M2 polarization markers on macrophages cultured with pentraxins. PBMC were cultured with either (**a**-**c**) 25 ng/ml M-CSF or (**d**-**f**) 25 ng/ml GM-CSF for 6 days and then SAP (10 μg/ml), CRP (10 μg/ml), or PTX3 (10 ng/ml) was added for an additional two days. PBMC were then air-dried, fixed, and stained by immunocytochemistry (ICC) with the indicated antibodies or irrelevant control antibodies. Following immunocytochemical staining, at least 100 macrophages were examined from at least 10 randomly selected fields, and the percentage of positive cells is expressed as the mean ± SEM (*n* = 4–5 separate donors)

**Fig. 2:**
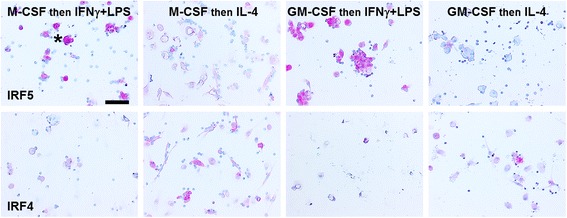
Expression of IRF5 (M1 marker) and IRF4 (M2 marker) on polarized macrophages. PBMC were cultured with 25 ng/ml of either M-CSF or GM-CSF for 6 days, and macrophages were then polarized for 2 days with either LPS + IFNγ or IL-4. Cells were then air-dried, fixed, and stained by ICC with antibodies. Positive cells are identified by red staining, and nuclei are counterstained blue. Bar is 100 μm. Asterisk indicates a cluster of macrophages stained with anti-IRF5 antibodies

There is no definitive set of markers to identify polarized macrophages [4, 5]. Therefore, we first assessed the effect of pentraxins on macrophage polarization using markers previously identified as being expressed on M1 macrophages including IFN-γ receptor (CD119), MHC class II, CCR7, and IRF5; M2a macrophages including Dectin-1, resistin-like molecule-β (RELMβ; FIZZ2), IRF4, and fibronectin (Fn); and M2c macrophages including sphingosine kinase-1 (SPK), TNFRSF14 (CD270) and SIRPα (CD172a) [4, 35–42]. As discussed by Murray et al. [4], all macrophages regulate the immune response, therefore we will use the term M2c, as defined by Mantovani [3], to define the IL-10 producing regulatory/resolving macrophage phenotype. We specifically did not isolate monocytes away from the other cell types found in PBMC, so that we could determine the effect of pentraxins and their ligands on monocyte differentiation and macrophage polarization in the presence of other immune cells. PBMC were cultured for 6 days in the presence of GM-CSF to differentiate the monocytes into M1-like macrophages, or M-CSF to differentiate the monocytes into M2-like macrophages [4, 33, 43, 44], and then pentraxins were added for additional 2 days to prime the macrophages. Compared to no pentraxin, there was no significant effect of SAP, CRP, or PTX3 on the percent of morphologically-identifiable macrophages expressing the markers listed above (Fig. 1). Antibodies against many of the markers only stained a subset of macrophages, indicating a heterogeneity of macrophages in these standard culture conditions. To confirm that we could identify polarized macrophages, PBMC were cultured for 6 days in the presence of M-CSF or GM-CSF and then polarized into M1 or M2 macrophages with either IFN-γ and LPS or IL-4. We found that the M1 marker IRF5 was upregulated in cultures containing IFN-γ and LPS, and the M2 marker IRF4 was upregulated in cultures containing IL-4 [33, 35, 36] (Fig. 2). These results suggest that pentraxins do not appear to affect the percent of macrophages expressing CD119, MHC class II, CCR7, IRF5, Dectin-1, RELMβ, IRF4, fibronectin, SPK, CD270, or CD172a.

**Fig. 3:**
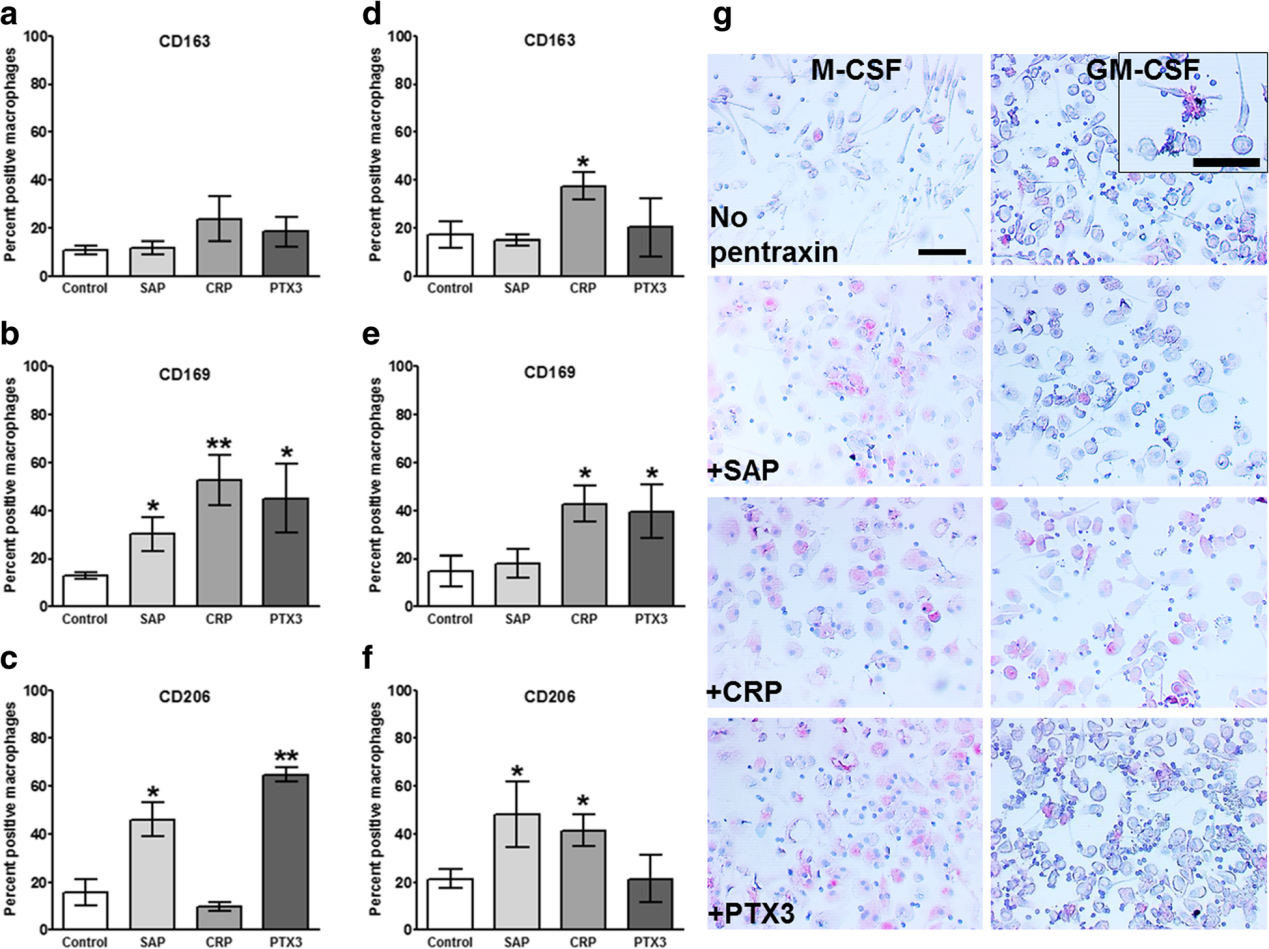
Effect of a single concentration of pentraxin on macrophage markers. PBMC were cultured with either (**a**-**c**) 25 ng/ml M-CSF or (**d**-**f**) GM-CSF for 6 days and then SAP (10 μg/ml), CRP (10 μg/ml), or PTX3 (10 ng/ml) was added for an additional two days. Cells were then air-dried, fixed, and stained by ICC with antibodies against (**a** and **d)** CD163, **b** and **e)** CD169, **c** and **f)** CD206. Results shows the percent positive macrophages expressed as the mean ± SEM (*n* = 3–4 separate donors). *p < 0.05, **p < 0.01 (1-way ANOVA with Dunn’s test). **g** Representative images of PBMC cultured in the presence or absence of pentraxins and then stained for CD169. Bar is 0.1 mm. Insert shows a dendritic cell in PBMC cultured in GM-CSF

Testing other markers, we then found that the hemoglobin-haptoglobin complex receptor CD163, the sialoadhesin CD169, and the C-type lectin CD206 were differentially expressed by human macrophages when cultured in the presence or absence of pentraxins (Fig. 3). CD163 has been used as both a tumor-associated and M2 macrophage marker, CD169 has been used as a marker of subsets of macrophages in lymph nodes, lung, and GI tract independent of the M1/M2 classification system, and CD206 has been used as a general M2 marker [4, 37, 45, 46]. However, the association of these three receptors with the broad M2 classification is difficult to interpret, as the M2 subset of macrophages contains pro-fibrotic M2a macrophages, M2c anti-inflammatory macrophages, and tumor-associated macrophages [4]. Compared to macrophages cultured with M-CSF in the absence of pentraxins, SAP increased the percentage of macrophages expressing CD169 and CD206, CRP increased the percentage expressing CD169, and PTX3 increased the percentage expressing CD169 and CD206 (Fig. 3a-c). Compared to macrophages cultured with GM-CSF in the absence of pentraxins, SAP increased the percentage expressing CD206, CRP increased the percentage expressing CD163, CD169, and CD206, and PTX3 increased the percentage expressing CD169 (Fig. 3d-f). We occasionally observed (by morphology) dendritic cells in cultures with GM-CSF (Fig. 3g insert). These results suggest that in cultures with M-CSF or GM-CSF, pentraxins affect the percentage of macrophages expressing detectable levels of CD163, CD169, and CD206.

### Effect of pentraxin ligands on macrophages

**Fig. 4:**
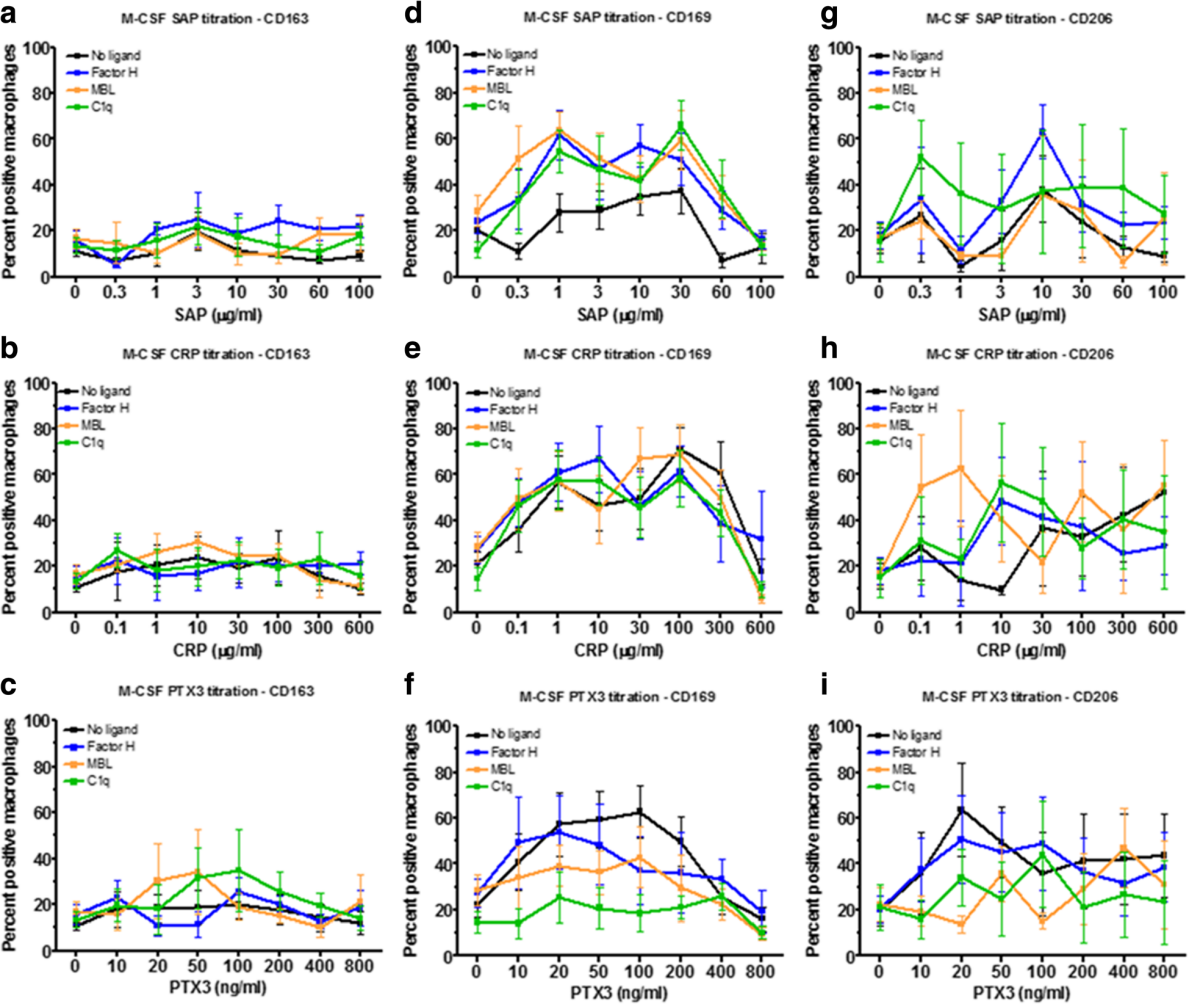
Effect of M-CSF priming, pentraxin concentration, and pentraxin ligands on macrophage markers. PBMC were cultured in M-CSF for 6 days and then with increasing concentrations of (**a**, **d**, **g**) SAP, (**b**, **e**, **h**) CRP, or (**c**, **f**, **i**) PTX3, in the presence or absence of factor H (100 μg/ml), MBL (2 μg/ml), or C1q (30 μg/ml), for an additional two days. Cells were then air-dried, fixed, and stained by ICC with antibodies against (**a**-**c**) CD163, (**d**-**f**) CD169, and **g**-**i**) CD206. Results shows the percent positive macrophages expressed as the mean ±SEM (*n* = 4 CD163; *n*= 9 CD169; *n*= 4 CD206 separate donors)

In healthy humans the plasma levels of CRP and PTX3 are low (<2 μg/ml and < 25 ng/ml respectively) and SAP is approximately 30 μg/ml, whereas during inflammation CRP and PTX3 levels may rise to 50–500 μg/ml and 200–800 ng/ml respectively, but SAP levels remain constant [7]. Pentraxins bind to several plasma proteins. SAP, CRP, and PTX3 all bind the complement component C1q [20–22], CRP and PTX3 bind Factor H, while SAP does not [7, 23], and SAP and PTX3, but not CRP, bind mannose-binding lectin (MBL) [24]. The plasma concentrations of C1q (50–200 μg/ml), Factor H (200–600 μg/ml), and MBL (1–3 μg/ml) are relatively constant and are not significantly altered during inflammation [47–51]. To determine if the above factors affect the response of macrophages to pentraxins, we cultured human PBMC with either M-CSF or GM-CSF for 6 days and then added increasing concentrations of pentraxins in the presence or absence of a single concentration of each pentraxin-binding ligand, and cultured the cells for an additional 2 days. For the cells cultured with M-CSF, neither the pentraxins nor the ligands had any significant effect on the percentage of macrophages expressing CD163 (Fig. 4a-c). 3 to 30 μg/ml SAP, 1 to 300 μg/ml CRP, and 20 to 200 ng/ml PTX3 increased the percentage of cells expressing CD169 (Fig. 4d-f). At 1 and 60 μg/ml SAP, all three ligands increased the percentage of macrophages expressing CD169. In the presence of CRP, the ligands had no significant effect, and in the presence of 20 to 200 ng/ml PTX3, C1q significantly reduced the percentage of macrophages expressing CD169. 10 μg/ml SAP, 30–600 μg/ml CRP (higher concentrations than used for the data in Fig. 3), and 20 to 800 ng/ml PTX3 increased the percentage of cells expressing CD206 (Fig. 4f-i). In the presence of 20 ng/ml PTX3, MBL reduced the percentage of macrophages expressing CD206 (Fig. 4i). These results suggest that for macrophages cultured with M-CSF, pentraxins and the ligands C1q and MBL can modulate the expression of CD169 and CD206.

**Fig. 5:**
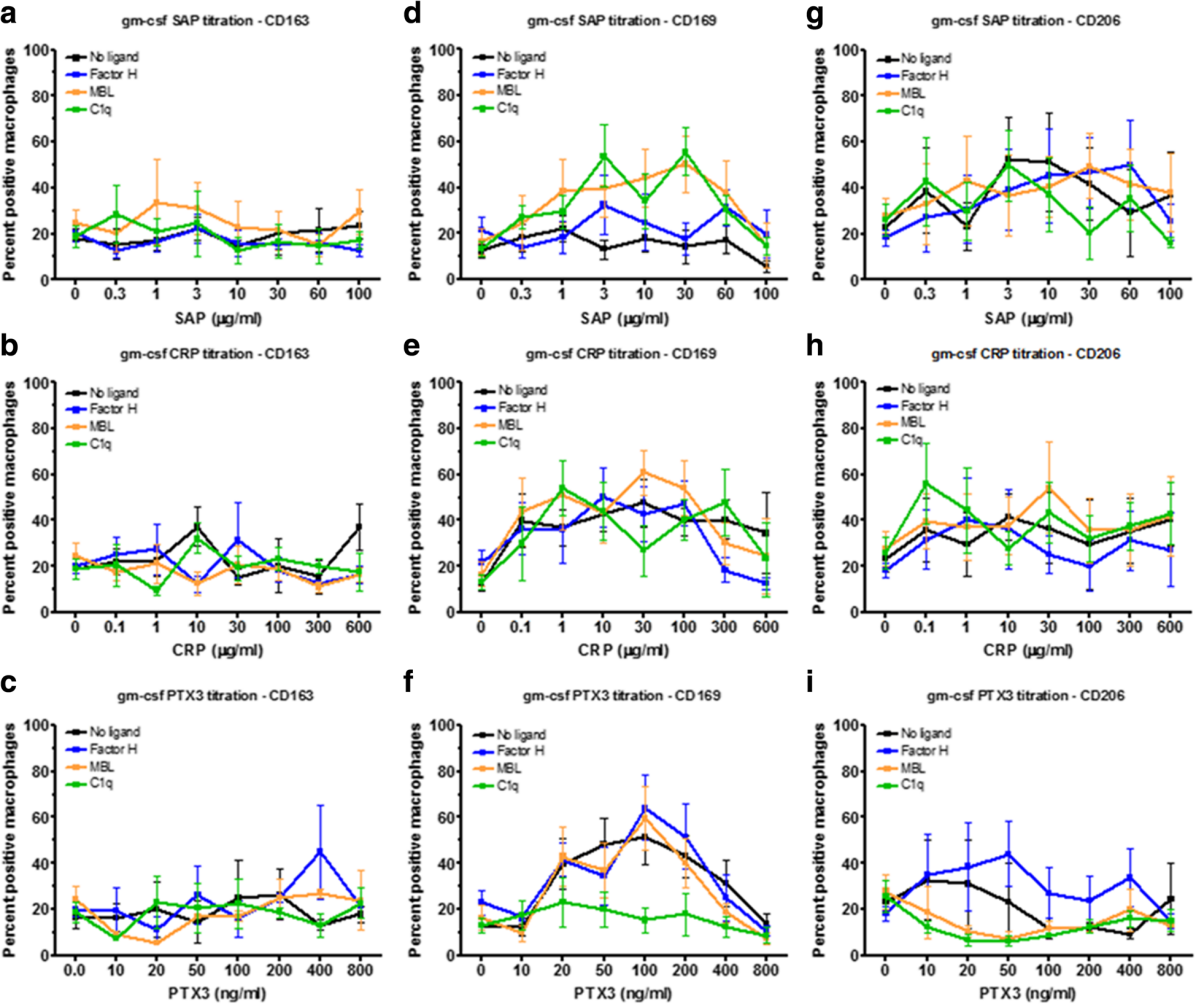
Effect of GM-CSF priming, pentraxin concentration, and pentraxin ligands on macrophage markers. PBMC were cultured in GM-CSF for 6 days and then with increasing concentrations of (**a**, **d**, **g**) SAP, (**b**, **e**, **h**) CRP, or **c**, **f**, **i**) PTX3, in the presence or absence of factor H (100 μg/ml), MBL (2 μg/ml), or C1q (30 μg/ml), for an additional two days. Cells were then air-dried, fixed, and stained by ICC with antibodies against (**a**-**c**) CD163, (**d**-**f**) CD169, and **g**-**i**) CD206. Results shows the percent positive macrophages expressed as the mean±SEM (*n* = 4 CD163; *n* = 9 CD169; *n* = 4 CD206 separate donors)

For the cells cultured with GM-CSF, we found that neither the pentraxins nor the ligands had any significant effect on the percentage of macrophages expressing CD163, apart from CRP at 10 μg/ml (Fig. 5a-c). 1 to 300 μg/ml CRP and 20 to 400 ng/ml PTX3 increased the percentage of cells expressing CD169 (Fig. 5d-f). At 3 to 30 μg/ml SAP, MBL and C1q increased the percentage of macrophages expressing CD169, in the presence of CRP the ligands had no effect, and at 50 to 200 ng/ml PTX3, C1q significantly reduced the percentage of macrophages expressing CD169. 3 and 10 μg/ml SAP and 10 μg/ml CRP increased the percentage of macrophages expressing CD206 (Fig. 5g). These results suggest that for macrophages cultured with GM-CSF, pentraxins and the ligands C1q and MBL can modulate the expression of CD169 and CD206.

### CRP can potentiate IL-10 accumulation

**Fig. 6:**
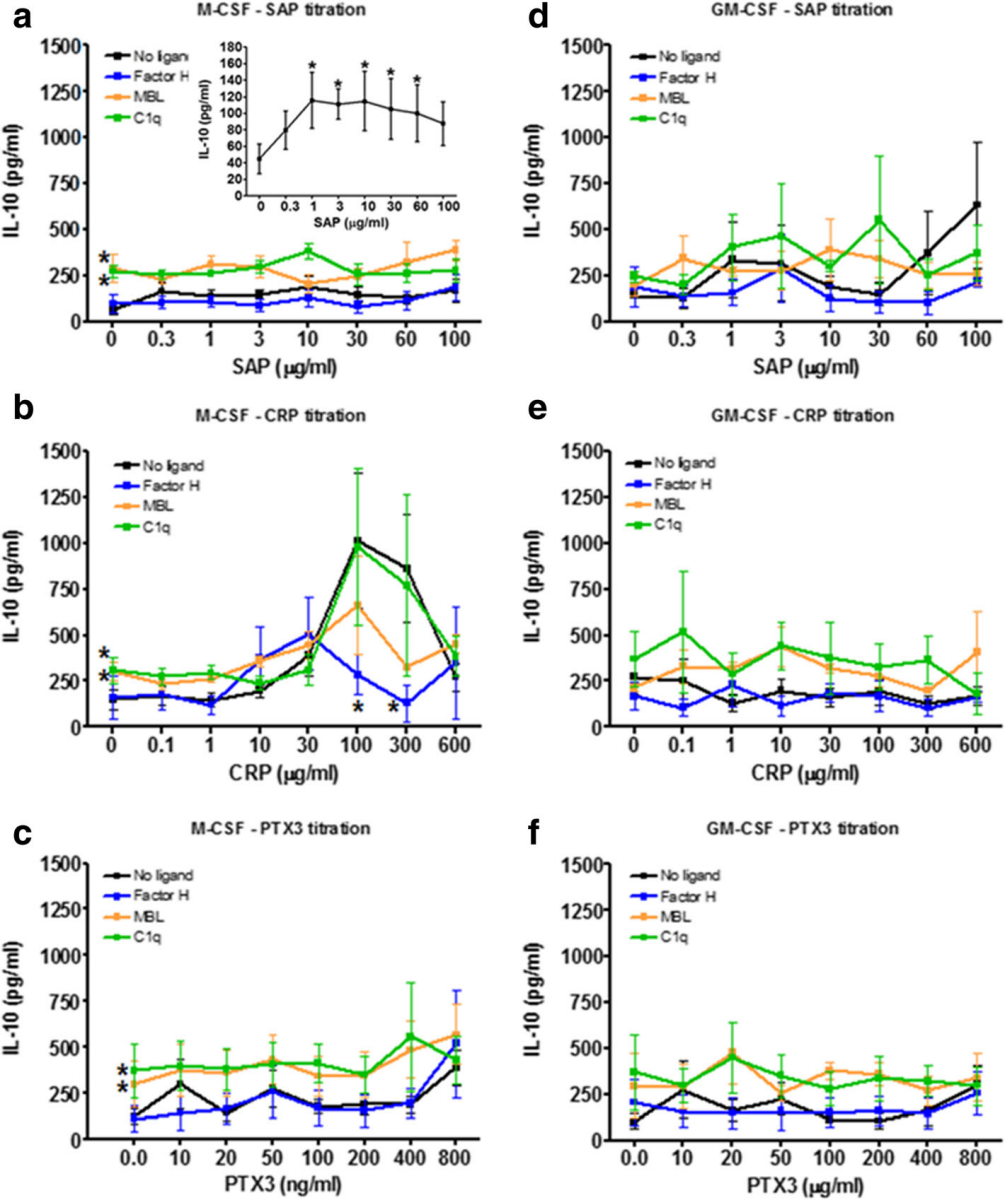
Effect of priming, pentraxin concentration and pentraxin ligands on IL-10 production. PBMC were cultured in (**a**-**c**) M-CSF or **d**-**f**) GM-CSF for 6 days and then with increasing concentrations of (**a**, **d**) SAP, **b**, **e**) CRP, or **c**, **f**) PTX3, in the presence or absence of factor H (100 μg/ml), MBL (2 μg/ml), or C1q (30 μg/ml), for an additional two days. Supernatants were then collected from the cells and assessed by ELISA for IL-10. Insert shows IL-10 production by SAP alone. Values are mean ± SEM (*n* = 8 SAP for M-CSF; *n* = 9 CRP for M-CSF; *n* = 9 PTX3 for M-CSF, *n* = 5 SAP for GM-CSF; *n* = 9 CRP for GM-CSF; *n* = 5 PTX3 for GM-CSF per group). *p < 0.05 (1-way ANOVA with Dunn’s test)

Besides cell surface receptors, M1 and M2 primed macrophages also secrete different cytokines, M1 macrophages secrete elevated levels of IL-12, M2a fibrotic macrophages secrete IL-4, and M2c macrophages secrete IL-10 [4, 6]. We collected supernatants from cells cultured in the presence of M-CSF and GM-CSF, and then primed with pentraxins in the presence or absence of ligands, and assayed for IL-4, IL-10, IL-12, and IFN-γ. We only found detectable levels of IL-10 in our culture conditions. As previously described [8], in the presence of M-CSF or GM-CSF, SAP increased IL-10 accumulation (Fig. 6a and d). In M-CSF, but not GM-CSF, 30 to 300 μg/ml CRP increased IL-10 accumulation (Fig. 6b). PTX3 had no significant effect on IL-10 accumulation. With M-CSF and no pentraxins, MBL and C1q increased IL-10 accumulation (Fig. 6a-c). With M-CSF and 100 and 300 μg/ml CRP, Factor H and MBL decreased IL-10 accumulation (Fig. 6b). These results suggest that CRP can affect IL-10 production and that this is reduced by the presence of GM-CSF, Factor H, or MBL.

To determine if the lack of signal for IL-12 was due to the pentraxins not stimulating the cells to secrete IL-12, or a technical issue with ELISA sensitivity, we tested the supernatants from M1- and M2-primed macrophages. When cells were cultured with M-CSF or GM-CSF and then polarized with LPS+ IFN-γ, we could detect high levels of both IL-10 and IL-12 (Additional file 1: Figure S1). These results suggest that the inability to detect IL-12 in cultures of PBMC with pentraxins and/or ligands indicates that these molecules do not generate a signal that induces IL-12 production.

### Effect of pentraxins and ligands on monocyte differentiation

**Fig. 7:**
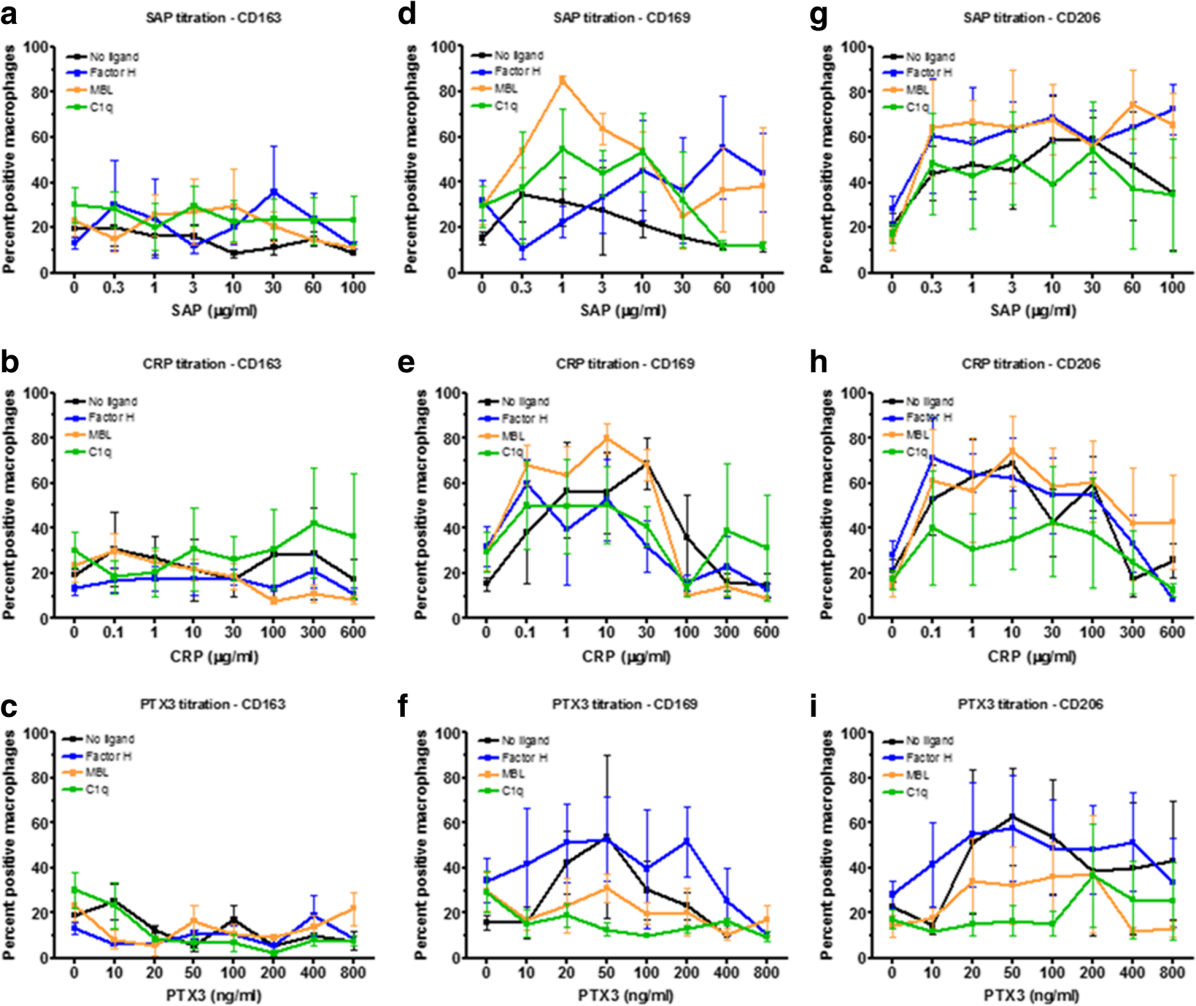
Effect of pentraxin concentration and pentraxin ligands on monocyte differentiation. PBMC were cultured in for 6 days with increasing concentrations of (**a**, **d**, **g**) SAP, (**b**, **e**, **h**) CRP, or **c**, **f**, **i**) PTX3 in the presence or absence of factor H (100 μg/ml), MBL (2 μg/ml), or C1q (30 μg/ml). Cells were then air-dried, fixed, and stained by ICC with antibodies against (**a**-**c**) CD163, (**d**-**f**) CD169, and (**g**-**i**) CD206. Results show the percent positive macrophages expressed as the mean±SEM (*n* = 4 separate donors)

Depending on the health of an individual, monocytes will be exposed to different concentrations of pentraxins in the blood and as they differentiate into macrophages in the tissues. To model these conditions, we cultured PBMC for 6 days to induce monocyte differentiation into macrophages in the presence or absence of pentraxins and ligands. None of the pentraxins had a significant effect on the percentage of macrophages expressing CD163 (Fig. 7a-c). 1 to 30 μg/ml CRP and 20 to 50 ng/ml PTX3 increased the percentage of macrophages expressing CD169 (Fig. 7e-f). 0.3 to 60 μg/ml SAP, 0.1 to 100 μg/ml CRP, and 10 to 400 pg/ml PTX3 increased the percentage of macrophages expressing CD206 (Fig. 7g-i). The ligands did not significantly alter the number of macrophages expressing CD163 (Fig. 7a-c). At 0.3 to 10 μg/ml SAP, MBL and C1q increased the percentage of macrophages expressing CD169 (Fig. 7d). At 20 to 50 ng/ml PTX3, MBL and C1q decreased the percentage of macrophages expressing CD169 (Fig. 7 f), and at 20 to 100 ng/ml PTX3, C1q also decreased the percentage of macrophages expressing CD206 (Fig. 7i). These results suggest that for monocytes differentiating into macrophages, pentraxins and the ligands C1q and MBL can modulate the expression of CD169 and CD206.

**Fig. 8:**
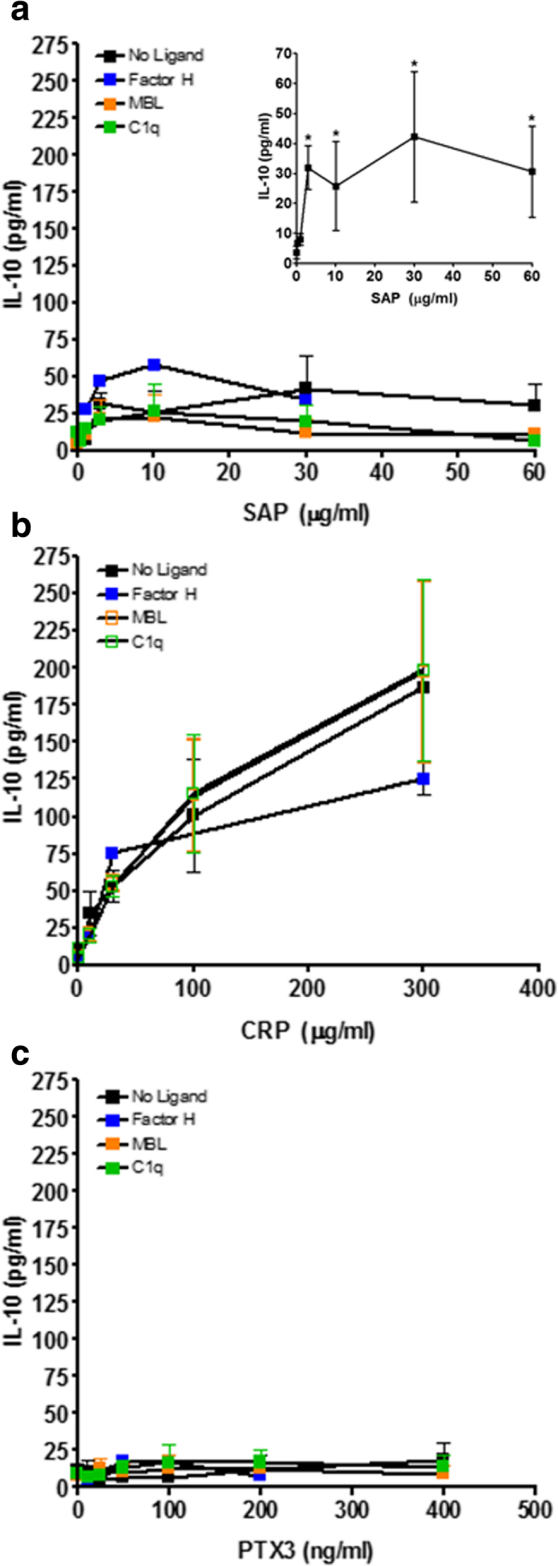
Effect of differentiation, pentraxin concentration, and pentraxin ligands on IL-10 production. PBMC were cultured for 6 days with increasing concentrations of (**a**) SAP, (**b**) CRP, or (**c**) PTX3, in the presence or absence of factor H (100 μg/ml), MBL (2 μg/ml), or C1q (30 μg/ml). Supernatants were then collected from the cells and assessed by ELISA for IL-10. Insert shows IL-10 production by SAP alone. Values are mean ± SEM (*n* = 3–4 separate donors). *p < 0.05 (1-way ANOVA with Dunn’s test)

To determine if pentraxins and/or their ligands also regulate extracellular cytokine accumulation as monocytes differentiate into macrophages, we collected supernatants from cells cultured in the presence of pentraxins in the presence or absence of ligands, and assayed for IL-4, IL-10, IL-12, and IFN-γ. We only found detectable levels of IL-10 in our culture conditions. As described previously [8], SAP modestly increased IL-10 accumulation (Fig. 8). CRP strongly potentiated IL-10 accumulation, and PTX3 had no significant effect. The addition of ligands did not significantly affect IL-10 accumulation (Fig. 8). These results suggest that for monocytes differentiating into macrophages, CRP can also affect IL-10 production and this is independent of ligands.

## Discussion

Pentraxins regulate macrophage responses, either by enhancing phagocytosis, by regulating complement activation, or by directly binding to receptors to alter macrophage differentiation and polarization [10, 11, 52]. In this report, we found that CD163, CD169, and CD206 expression was differentially regulated by pentraxins, and that the pentraxin ligands Factor H, MBL, and C1q altered some of these responses for CD169 and CD206. In addition, we found that CRP was a potent inducer of IL-10 production in monocytes and macrophages cultured in the presence of M-CSF but not GM-CSF.

We found that most of the published macrophage polarization markers were unaltered by culturing cells in the presence of the pentraxins, even though these same markers were expressed differentially by macrophages using standard polarization conditions. These data suggest that pentraxin regulation of macrophage polarization is more subtle than a straightforward M1/M2a/M2c scheme and more akin to the view of macrophage polarization as a continuum[5]. In addition, we found that the presence of Factor H, MBL, and C1q altered the expression of macrophage markers induced by pentraxins, such that C1q augmented the expression of CD169 by SAP, but C1q inhibited CD169 and CD206 expression induced by PTX3. Several groups including our own have previously shown that SAP and PTX3 can promote CD206 expression, but the observation that MBL and C1q can counteract these effects again suggests that experiments with a single pentraxin concentration do not adequately represent the environment found at sites of inflammation.

CRP is generally thought of as being an inflammatory mediator, due to its upregulation during infection and the correlation of high CRP levels with poor prognosis in persistent inflammatory conditions such as cardiovascular disease [14]. However, others have argued that the effect of CRP is more subtle and the concentration of CRP present in a lesion, the presence of co-factors such as bacterial products and complement pathway proteins, and the site of tissue response may determine the pro- or anti-inflammatory nature of CRP [7, 17]. Several reports also indicate that CRP can promote the production of the anti-inflammatory cytokine IL-10, suggesting that elevated CRP levels may by a means to downregulate inflammation [17, 18, 53, 54]. The effect of CRP may be further complicated by the relative levels of CRP in the circulation compared to the tissue or inflammatory site, as transgenic mice expressing CRP in lesions have differential responses to mice with high levels of systemic CRP [55].

The role of PTX3 in regulating inflammation is also dependent on spatial and temporal conditions [7]. PTX3 can reduce platelet activation and neutrophil migration during the early stages of inflammation, and bind complement component proteins (such as C1q, MBL, and Factor H), to limit tissue injury [56, 57]. However, increased PTX3 levels can exacerbate persistent and autoimmune diseases, such as chronic heart and lung diseases [58–60]. Our observations that MBL and C1q can reverse PTX3-induced CD169 and CD206 expression suggest that both local and systemic concentrations of pentraxin ligands will have a profound effect on macrophage phenotype and function.

The four proteins regulated by pentraxins were the hemoglobin-haptoglobin complex receptor CD163, the surface receptors CD169 and CD206, and the anti-inflammatory cytokine IL-10. CD163 is a member of the scavenger receptor cysteine-rich (SRCR) superfamily, and is exclusively expressed in monocytes and macrophages [61]. CD163 is a receptor involved in the clearance and endocytosis of hemoglobin/haptoglobin complexes by macrophages, and may thereby protect tissues from free hemoglobin-mediated oxidative damage [62]. CD163 expression is upregulated by glucocorticoids and IL-10, and downregulated by LPS, TNF, and GM-CSF, suggesting that CD163 is a marker for alternatively activated macrophages [63, 64]. However, CD163 positive macrophages are frequently found in tissue samples from chronic inflammation, and high levels of soluble CD163 are present in plasma from a wide range of inflammatory diseases [65–67].

CD169, also known as Sialoadhesin or Siglec-1, is a lectin that binds to proteins with sialic acid residues, and is expressed by subsets of macrophages in secondary lymphoid organs (spleen and lymph nodes) and in tissues exposed to environmental antigens (lung, GI tract, and liver) [46]. CD169 appears to promote the phagocytosis of pathogens, leading to enhanced immune responses, but inhibits autoimmune responses [68]. However, increased CD169 expression promotes macrophage uptake of pathogens to augment adaptive T cell and B cell responses, but increased CD169 is also associated with an increased risk of autoimmune and cardiovascular disease [46, 69, 70]. These data suggest that the local and systemic concentrations of SAP, PTX3, C1q, and MBL will ultimately regulate CD169 expression and function.

CD206, also known as the macrophage mannose receptor is a lectin that binds to mannose, N-acetylglucosamine, and fucose sugars on molecules, but only in the presence of calcium [71]. CD206 is also expressed by specific subsets of macrophages, including lung alveolar macrophages and spleen, lymph node and bone marrow macrophages, but in different anatomical sites to macrophages expressing CD169 [72]. CD206 recognition of bacteria without bound complement components (unopsonized) suppresses macrophage activation, whereas macrophage activation does occur when bacteria are opsonized and therefore bind receptors on macrophages other than CD206 [73–75]. This appears to be a mechanism to prevent inflammatory responses against commensal bacteria, such as in in the lung [42].

The expression of CD163, CD169, and CD206 on monocyte/macrophages appears to be regulated by a variety of factors including cytokines, with interferons and TNFα preventing or downregulating expression, and IL-4 and IL-10 upregulating expression of these 3 receptors [46, 76, 77]. In addition, CD169 binds sialic acid residues, and CD206 binds mannose residues on pentraxins [7], and both receptors appear to interact with Fc receptors to regulate Fc receptor signaling, internalization, and recycling [46, 78]. Therefore, pentraxins may regulate the expression of these three receptors either by altering the cytokine milieu and/or by directly binding to the receptors, and the presence of the ligands may alter these processes.

IL-10 is an anti-inflammatory cytokine released by many cells, including macrophages and epithelial cells, in response to Fcγ receptor and CD209 (DC-SIGN) activation by IgG, SAP, and CRP [8, 11, 17, 53, 79]. In macrophages, the production of IL-10 appears to be dependent on FcγR ligation, leading to ERK activation, which in turn causes remodeling of the chromatin at the IL-10 locus, making it more accessible to transcription factors [80]. In IL-10 knockout mice, the protective effects of CRP and SAP on inflammation, nephritis, EAE, and lung fibrosis is reduced or absent, suggesting that these systemic pentraxins can act to quench ongoing inflammatory responses [11, 17, 18, 53, 55]. As SAP, in most animals, is relatively constant and CRP is the acute phase response proteins (whereas in mice the situation is revered), these data also suggest that the two pentraxins may cooperate to regulate inflammation [7]. The situation with PTX3 is different, as PTX3 does not appear to stimulate IL-10 production, but PTX3 production is stimulated by IL-10 [7]. This suggests that the upregulation of PTX3 following inflammation may in part be modulated by SAP and CRP-induced IL-10 production, suggesting a feedback loop between the three pentraxins.

In health, the systemic levels of Factor H, MBL, and C1q are relatively constant [47–51]. However, during inflammation the local activation of complement and the presence of bacteria and cell debris can lead to a local reduction in Factor H, MBL, and C1q levels, whereas the levels of CRP and PTX3 may increase (due to systemic and local production) at these same sites [7, 81]. In addition, either a genetic deficiency of C1q or Factor H, or reduced serum concentrations due to increased consumption and/or neutralization by autoantibodies, leads to activated macrophages and is a major susceptibility factor for the development of systemic lupus erythematosus (SLE) [82–84]. Similar MBL deficiencies lead to increased infection by influenza and exaggerated macrophage activation and increases in inflammatory cytokines, such as IL-1β and TNFα [85–87].

## Conclusion

Together, our results suggest that the levels of pentraxins and their ligands affect monocyte differentiation and macrophage priming, and that as CRP is a potent inducer of the anti-inflammatory cytokine IL-10, elevated levels of this pentraxin may not always be associated with pro-inflammatory responses.

## Additional file

Additional file 1: Figure S1. Effect of standard polarization conditions on IL-10 and IL**-**12 production PBMC were cultured with either 25 ng/ml M-CSF or GM-CSF for 6 days and then polarized for 2 days with either LPS + IFNγ or IL-4. Supernatants were then collected from the cells and tested by ELISA for IL-10 and IL-12. Values are mean ± SEM, *n* = 3. (TIF 107 kb)

## Abbreviations

CRP: C-reactive protein
EAE: Experimental allergic encephalomyelitis
FcγR: Fcγ receptors
GM-CSF: Granulocyte Macrophage colony stimulating factor
ICC: Immunocytochemistry
MBL: Mannose-binding lectin
M-CSF: Macrophage colony stimulating factor
PBMC: Peripheral blood mononuclear cells
PTX3: Pentraxin-3
SAP: Serum amyloid P
