# Supplementary figures and images for: Monocyte differentiation and macrophage priming are regulated differentially by pentraxins and their ligands
Source: BMC Immunol. 2017 Jun 15;18:30. doi: 10.1186/s12865-017-0214-z (PMC5472910; doi:10.1186/s12865-017-0214-z)

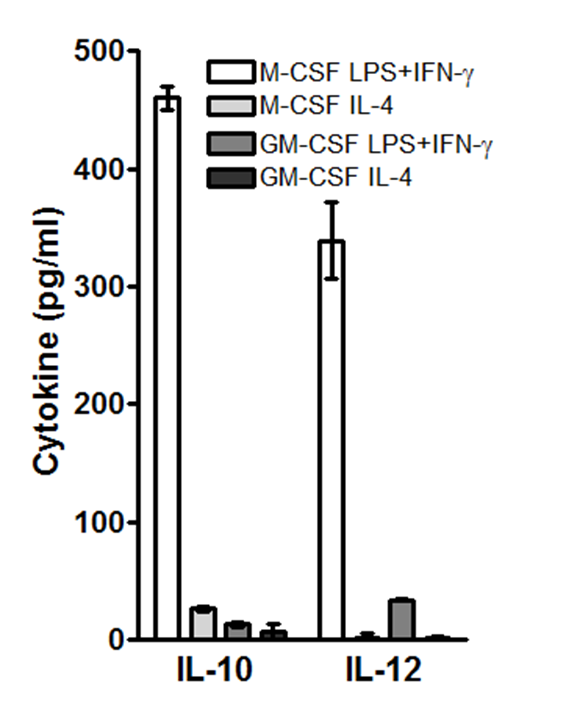

Supplement: Additional file 1: Figure S1. — Effect of standard polarization conditions on IL-10 and IL-12 production PBMC were cultured with either 25 ng/ml M-CSF or GM-CSF for 6 days and then polarized for 2 days with either LPS + IFNγ or IL-4. Supernatants were then collected from the cells and tested by ELISA for IL-10 and IL-12. Values are mean ± SEM, n = 3. (TIF 107 kb) [file 12865_2017_214_MOESM1_ESM.tif]
